# Significance of AT_1_ Receptor Independent Activation of Mineralocorticoid Receptor in Murine Diabetic Cardiomyopathy

**DOI:** 10.1371/journal.pone.0093145

**Published:** 2014-03-24

**Authors:** Yuji Nagatomo, Tomomi Meguro, Hiroyuki Ito, Kimi Koide, Toshihisa Anzai, Keiichi Fukuda, Satoshi Ogawa, Tsutomu Yoshikawa

**Affiliations:** 1 Cardiology Division, Department of Medicine, Keio University School of Medicine, Tokyo, Japan; 2 Department of Cardiology, KAKEN Hospital, International University of Health and Welfare, Ichikawa, Japan; Cleveland Clinic Lerner Research Institute, United States of America

## Abstract

**Background:**

Diabetes mellitus (DM) has deleterious influence on cardiac performance independent of coronary artery disease and hypertension. The objective of the present study was to investigate the role of the renin-angiotensin-aldosterone system, especially angiotensin II type 1a receptor (AT_1a_R) and mineralocorticoid receptor (MR) signaling, in left ventricular (LV) dysfunction induced by diabetes mellitus (DM).

**Methods and Results:**

DM was induced by intraperitoneal injection of streptozotocin (200 mg/kg BW) in wild-type (WT) or AT_1a_R knockout (KO) male mice, and they were bred during 6 or 12 weeks. Some KO mice were administered the MR antagonist eplerenone (100 mg/kg body weight). At 6 weeks, LV diastolic function was impaired in WT-DM, but preserved in KO-DM. At that time point MR mRNA expression was upregulated, NADPH oxidase subunit (p47phox) and glutathione peroxidase (GPx1) mRNA expression were upregulated, the staining intensities of LV tissue for 4-hydroxy-2-nonenal was stronger in immunohistochemistry, the number of terminal deoxynucleotidyl transferase-mediated dUTP nick-end labeling (TUNEL) positive cells was increased, Bcl-2 protein expression was significantly downregulated, and the expression of SERCA2a and phosphorylated phospholamban was depressed in WT-DM, while these changes were not seen in KO-DM. At 12 weeks, however, these changes were also noted in KO-DM. Eplerenone arrested those changes. The plasma aldosterone concentration was elevated in WT-DM but not in KO-DM at 6 weeks. It showed 3.7-fold elevation at 12 weeks even in KO-DM, which suggests “aldosterone breakthrough” phenomenon. However, the aldosterone content in LV tissue was unchanged in KO-DM.

**Conclusions:**

DM induced diastolic dysfunction was observed even in KO at 12 weeks, which was ameliorated by minelarocorticoid receptor antagonist, eplerenone. AT_1_-independent MR activation in the LV might be responsible for the pathogenesis of diabetic cardiomyopathy.

## Introduction

Cardiovascular complications including coronary artery disease are a major cause of morbidity and mortality in patients with diabetes mellitus (DM). In addition, the risk of heart failure is two-fold higher in men with DM and five-fold higher in women with DM, according to the Framingham study [1], and DM is an independent risk factor for the occurrence of heart failure (HF) [2]. The presence of DM or even impaired glucose tolerance (IGT) in patients with HF has also been shown to be an independent risk factor for adverse outcome such as rehospitalization for HF [3]. A lot of studies have suggested that DM per se has an adverse effect on cardiac function [4], [5]. For example, left ventricular (LV) systolic and diastolic dysfunction occurs in rodents with streptozotocin-induced DM [5]. In patients with DM, diastolic dysfunction characteristically occurs first and is followed by impairment of contractility [6]. The molecular mechanisms underlying cardiac dysfunction related to DM include impaired calcium handling [7], increased oxidative stress [8]–[10], and an increase of apoptosis [9].

The renin-angiotensin-aldosterone system (RAAS) has an important role in the onset and progression of DM-associated vascular complications and DM-induced cardiac dysfunction, with the detrimental effect of angiotensin II type 1 receptor (AT_1_) signaling having attracted much attention [4], [11]. On the other hand, angiotensin II potently promotes aldosterone production [12]. Interruption of AT_1_ signaling by treatment with angiotensin-converting enzyme inhibitors (ACE-I) or angiotensin receptor blockers (ARB) results in a reduction of plasma aldosterone concentration (PAC), followed by a return to baseline during *long-term* administration [13], [14] that is referred to as “aldosterone breakthrough”. In this setting, mineralocorticoid receptor (MR) activation might play an important role in the deterioration of HF when patients are on chronic ACE-I or ARB therapy. It was shown that MR antagonist spironolactone provided additional beneficial effects on LV morphology and function to ARB in experimental myocardial infarction (MI) model rats [15]. In large-scale clinical trials, RALES [16], EPHESUS [17], EMPHASIS-HF [18] and Aldo-DHF study [19], addition of an MR blocker to standard medical therapy including ACE-I or ARB had a beneficial effect on the prognosis of patients with HF or MI.

However, it is still unknown whether the blocking of AT_1_ signaling can prevent DM-induced LV dysfunction over the *long-term*. If it couldn't, AT_1_-independent MR activation (including aldosterone breakthrough) might have an influence on DM-induced LV dysfunction. Accordingly, the goal of the present study was to determine whether blockade of AT_1_ signaling prevented DM-induced LV dysfunction over both the *short-term* and *long-term*. The second goal of this study was to elucidate whether AT_1_-independent MR activation has a role in the occurrence of DM-induced LV dysfunction.

## Materials and Methods

### Experimental Animals

**Figure 1:**
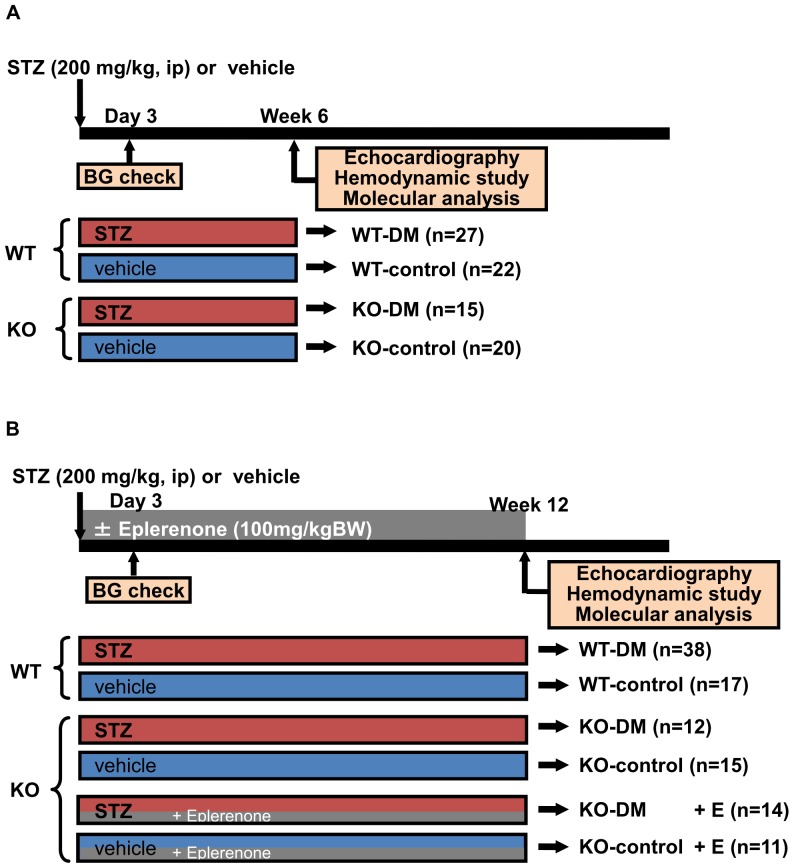
Experimental design. Six-week model (A) and 12-week model (B). STZ, streptozotocin; BG, blood glucose; DM, diabetes mellitus; KO, AT_1a_ receptor knockout; WT, wild-type.

Nine-week-old male mice with knockout of the angiotensin II type 1a receptor (AT_1a_R KO) were generated (KO mice) [20] and age-matched male mice without AT_1a_R KO that had the same genetic background (wild-type [WT] mice) were used as controls. The study design is shown in **Figure 1**. Diabetes was induced by a single intraperitoneal injection of streptozotocin (STZ, 200 mg/kg; Sigma-Aldrich, Tokyo, Japan), as described previously [21]. Streptozotocin (STZ) was dissolved in sterile sodium citrate buffer. In the control groups, citrate buffer alone was injected. Some KO mice were administered the MR antagonist eplerenone (100 mg/kg BW, Pfizer Inc., NY, USA) for 12 weeks. STZ-treated WT mice (WT-DM), STZ-treated KO mice (KO-DM), vehicle-treated WT mice (WT-control), and vehicle-treated KO mice (KO-control) were raised for 6 or 12 weeks, while STZ-treated KO mice with eplerenone therapy (KO-DM+E) and vehicle-treated KO mice with eplerenone therapy (KO-control+E) were raised for 12 weeks (6w: WT-DM n = 27, WT-control n = 22, KO-DM n = 15, KO-control n = 20. 12w: WT-DM n = 38, WT-control n = 17, KO-DM n = 12, KO-control n = 15, KO-DM+E n = 14, KO-control+E n = 11).

Plasma glucose concentrations were measured with a commercial kit (The Glucose Vision Blood Glucose Monitoring System; Adventure Healthcheck, CA, USA) at 3 days after the injection of STZ or vehicle.

### Ethics Statement

This study conformed with the *Guide for the Care and Use of Laboratory Animals* published by the US National Institutes of Health (NIH Publication No. 85–23, revised 1996), and approval was granted by the ethical review board of Keio University.

### Echocardiography

At 6 or 12 weeks after injection of STZ or the vehicle, echocardiography (En Visor C M2540A, Philips, Tokyo, Japan) was performed after anesthesia was induced with a single intraperitoneal injection of ketamine (70 mg/kg) and xylazine (7 mg/kg), as described previously [22]. The heart rate was monitored by electrocardiography in order to evaluate the adequacy of anesthesia. The average of three to four measurements was calculated for each parameter.

### Hemodynamic Study

At 6 or 12 weeks after injection of STZ or the vehicle, a 1.4-F micromanometer catheter (Millar Instruments, Inc., TX, USA) was inserted into the LV of each mouse via the right carotid artery under the above-mentioned anesthesia, while the heart rate was monitored by electrocardiography in order to evaluate the adequacy of anesthesia. Then LV pressure curves were recorded and hemodynamic data were obtained, as described previously [23]. Thereafter, the mice were euthanized by an overdose of ketamine and xylazine, their hearts were quickly excised and weighed, and peripheral blood was collected.

### Measurement of Aldosterone and Corticosterone

The PAC and plasma corticosterone concentration were determined with a commercially available kit (Aldosterone EIA Kit, Assay Designs, Inc., MI, USA, Corticosterone EIA Kit, Cayman Chemical Company, MI, USA). The corticosterone or aldosterone content of LV tissue was determined by liquid chromatography–electrospray ionization tandem mass spectrometry (LC–ESI-MS/MS), which has sufficient accuracy and precision to measure aldosterone and corticosterone with a high reliability and reproducibility [24].

### Real-time PCR

Total cellular RNA was extracted from LV tissue using TRIzol reagent (Invitrogen, CA, USA) as described previously [25]. cDNA was synthesized from total RNA using a High-capacity cDNA archive kit (Applied Biosystems, CA, USA). Real-time PCR was performed with an ABI 7500 Fast real-time PCR system (Applied Biosystems, CA, USA), as described previously [26]. Reaction mixtures containing each set of specific Taqman probes and primers (Applied Biosystems, CA, USA) were added to diluted samples of cDNA. Glyceraldehyde-3-phosphate dehydrogenase (GAPDH) was amplified as the internal control.

### Western Blotting

After LV myocardial tissues were homogenized in lysis buffer (T-PER Tissue Protein Extraction Reagent, PIERCE, IL, USA), samples were centrifuged and the supernatant was collected as the protein extract. Western blot analysis was performed using a commercially available antibody and proteins were detected with ECL Plus Western Blotting Detection Reagents (GE Healthcare UK Ltd. Buckinghamshire, UK).

### Pathological Examination

Excised LV tissues were fixed in 10% formalin and embedded in paraffin, as described previously [27]. Immunohistochemical staining of 4-hydroxy-2-nonenal (4-HNE) was performed with a commercially available kit (Histofine Mouse Stain Kit, Nichirei, Tokyo, Japan). Mouse monoclonal anti-HNE-modified protein antibody (1∶50 dilution, NOF Medical Department, Tokyo, Japan) was used as the primary antibody. Samples were examined under a light microscope [28]. Apoptosis was quantified by the terminal deoxynucleotidyl transferase-mediated dUTP nick-end labeling (TUNEL) method, as described previously [29]. Sections were stained by using a CardioTACS *In Situ* Apoptosis Detection Kit (Trevigen, Gaithersburg, MD, USA) according to the manufacturer's instructions. Twenty photographs (magnification ×200) were taken of each section. Then the nuclei of cardiomyocytes were manually counted by an observer who was blinded to the experimental conditions.

### Statistical Analysis

Data are expressed as the mean ± SEM. Multiple comparisons were performed by 1-way analysis of variance, followed by the Tukey-Kramer test. A probability <0.05 was considered to indicate statistical significance.

## Results

### Blood Glucose and Organ Weights

At 6 and 12 weeks, blood glucose levels were higher in all of the DM groups compared with the respective control groups. There were no differences of blood glucose levels among the DM groups. LV weight corrected by body weight was unchanged in all DM groups compared with the respective control groups (**Table 1**).

**Table 1:** Blood glucose level and morphology.

	6w				12w					
	WT		KO		WT		KO			
	control	DM	control	DM	control	DM	control	DM	control+E	DM+E
	(n = 20)	(n = 20)	(n = 20)	(n = 15)	(n = 17)	(n = 38)	(n = 15)	(n = 12)	(n = 11)	(n = 11)
Blood Glucose(mg/dl)	176±6	521±15*	163±7	477±28*	218±10	540±14^**^	186±12	510±33 ^**^	221±22	572±17 ^**^
BW (g)	27.0±0.3	20.0±0.4*	31.0±0.5	25.9±0.8*	29.5±0.5	21.1±0.5^**^	33.7±0.5	26.8±0.8^**^	34.8±1.1	24.6±1.2^**^
LV weight (mg)	82.7±1.1	62.0±1.6*	86.8±2.0	71.0±2.6*	93.6±2.1	68.2±2.0^**^	94.2±2.0	75.6±2.8^**^	89.0±3.1	65.9±4.8^**^
LV/BW (mg/g)	3.1±0.0	3.0±0.1	2.8±0.1	2.7±0.1	3.1±0.1	3.2±0.1	2.8±0.1	2.8±0.1	2.6±0.1	2.6±0.1

Values are expressed as mean±SEM. WT, wild-type mice; KO, AT_1a_ receptor knockout mice; E, eplerenone; BW, body weight; LV, left ventricle. *p<0.05 compared with respective control groups, **p<0.01 compared with respective control groups.

### Cardiac Function

#### LV systolic function

At 6 weeks, fractional shortening (FS) showed no significant differences among the 4 groups. With respect to the rate-corrected velocity of circumferential fiber shortening (Vcfc), however, the WT-DM group showed a significant reduction compared with the WT-control group, but the KO-DM group showed no significant change. Hemodynamic assessment revealed that the LV systolic pressure and peak positive dP/dt were similar among the four groups at 6 weeks.

**Figure 2:**
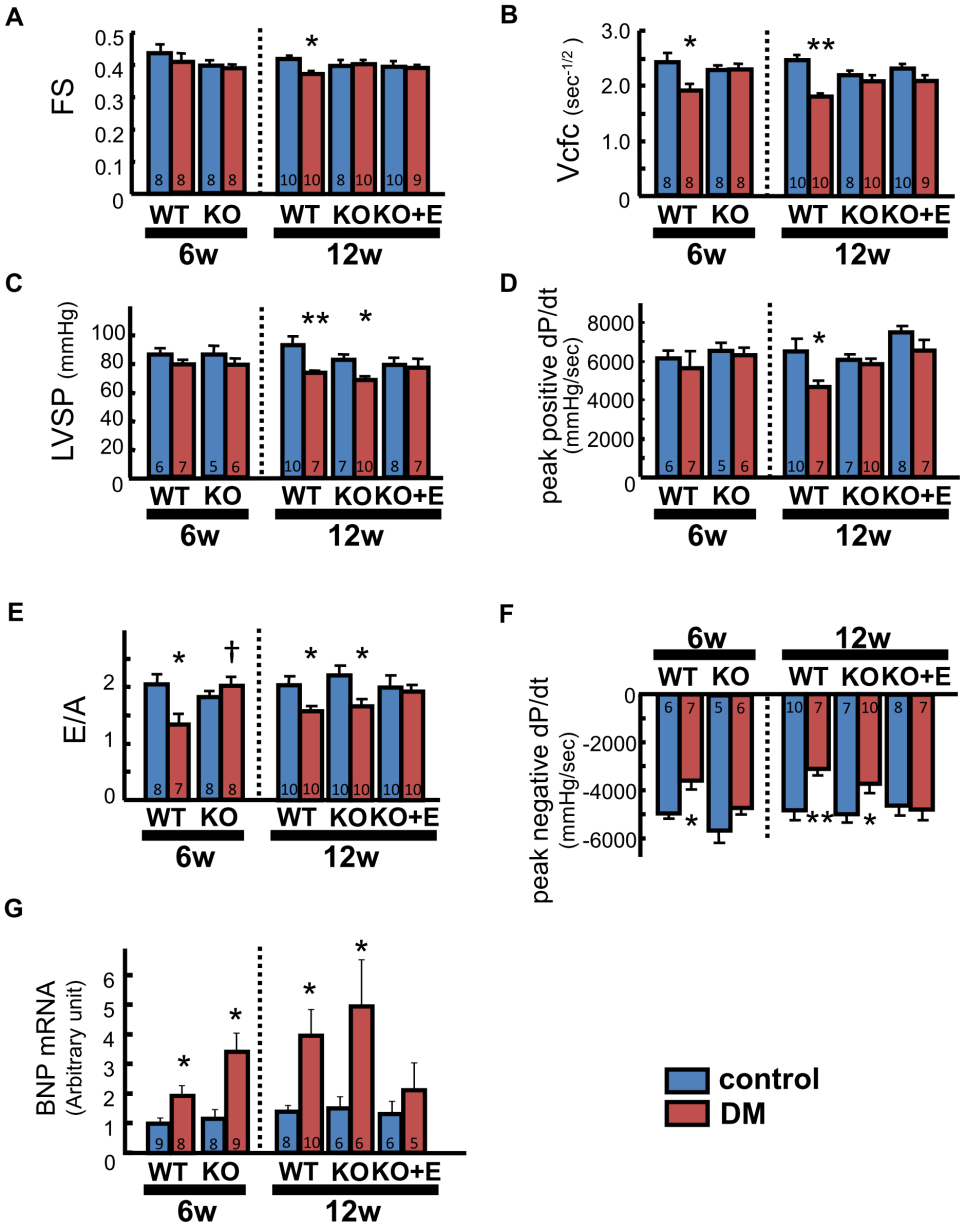
Indices of LV function. Fractional shortening (FS) (A), Vcfc (B), LV systolic pressure (LVSP) (C), peak positive dP/dt (D), E/A ratio (E), peak negative dP/dt (F), and brain natriuretic peptide (BNP) mRNA expression in LV tissue measured by real-time PCR (G). Number of mice per group is indicated in bar graph. Number of mice per group is indicated in the bar graph. Number of mice per group is indicated in the bar graph. Number of mice per group is indicated in the bar graph. Values are the mean±SEM. DM, diabetes mellitus; KO, AT_1a_ receptor knockout; WT, wild-type.*p<0.05 vs. the respective control group. **p<0.01 vs. the respective control group. †p<0.05 vs. WT-DM.

At 12 weeks, both FS and Vcfc were significantly lower in WT-DM mice compared with WT-control mice. However, there were no significant differences of these indices between the KO-DM and KO-control groups (**Figure 2A, 2B**). At 12 weeks, LV systolic pressure was significantly lower in the WT-DM and KO-DM groups compared with the respective control groups. However, there was no significant difference of LV systolic pressure between the KO-DM+E and KO-control+E groups. Peak positive dP/dt was significantly lower in WT-DM mice compared with WT-control mice. However, there were no significant differences of peak positive dP/dt among the KO groups (**Figure 2C, 2D**).

#### LV diastolic function

At 6 weeks, the E/A ratio calculated from pulsed-wave Doppler measurements of LV inflow and peak negative dP/dt (indices of LV diastolic function) were significantly lower in the WT-DM group compared with the WT-control group. In contrast, there were no significant differences of the E/A ratio or peak negative dP/dt among the KO groups.

At 12 weeks, however, the E/A ratio and peak negative dP/dt were also lower in the KO-DM group compared with the KO-control group. There were no significant differences of the E/A ratio or peak negative dP/dt between the KO-DM+E and KO-control+E groups (**Figure 2E, 2F**). There were no significant differences of heart rate or LV end-diastolic pressure among these groups at any time (data not shown).

#### LV tissue level of BNP mRNA

Brain natriuretic peptide (BNP) mRNA levels were significantly elevated in WT-DM and KO-DM mice compared with the respective control groups at both 6 and 12 weeks. However, the BNP mRNA level was similar in KO-DM+E and KO-control+E mice (**Figure 2G**).

### Ca^2+^ Handling

**Figure 3:**
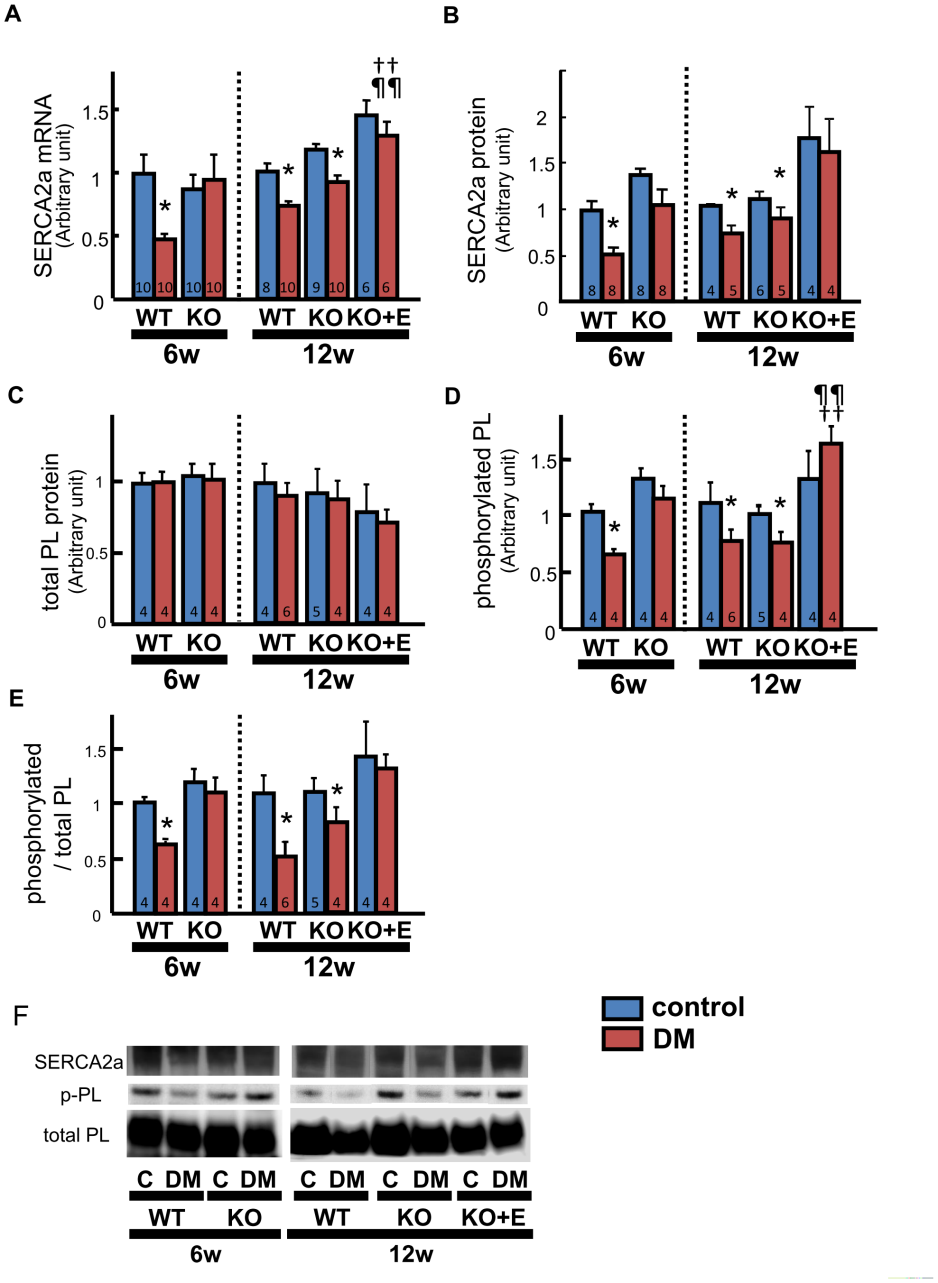
Ca^2+^ handling in the diabetic heart. Sarcoplasmic reticulum Ca^2+^-ATPase (SERCA2a) mRNA expression in LV tissue measured by real-time PCR (A), SERCA2a protein level (B), the contents of total (C) and phosphorylated (D) phospholamban protein in LV tissue determined by Western blotting, the ratio of phosphorylated to total phospholamban (E), and representative images of Western blots (F). Number of mice per group is indicated in bar graph. Values are the mean±SEM. DM, diabetes mellitus; KO, AT_1a_ receptor knockout; WT, wild-type; PL, phospholamban; C, control. *p<0.05 vs. the respective control group. ††p<0.01 vs. WT-DM, ¶¶p<0.01 vs. KO-DM.

At 6 weeks, sarcoplasmic reticulum Ca^2+^-ATPase (SERCA2a) mRNA and protein expression were downregulated in the WT-DM group compared with the WT-control group. (**Figure 3A, 3B**). However, there were no significant differences of SERCA2a mRNA and protein levels between the KO groups. At 12 weeks, SERCA2a mRNA and protein levels were also downregulated in KO-DM mice compared with KO-control mice. Eplerenone treatment prevented these changes (**Figure 3A, 3B**).

Phospholamban mRNA expression was similar among all groups at both 6 and 12 weeks (data not shown). Western blotting revealed that the total phospholamban protein content was similar among all groups (**Figure 3C**). At 6 weeks, the level of phosphorylated phospholamban was significantly reduced in WT-DM mice compared with WT-control mice, although there were no significant differences between the KO groups. At 12 weeks, however, phosphorylated phospholamban levels were also reduced in KO-DM mice compared with KO-control mice. Eplerenone treatment prevented these changes (**Figure 3D**). The ratio of phosphorylated phospholamban to total phospholamban was significantly lower in WT-DM mice than WT-control mice, but the KO groups showed no significant difference at 6 weeks. At 12 weeks, it was also lower in KO-DM mice compared with KO-control mice. Eplerenone treatment prevented these changes (**Figure 3E**).

### Apoptosis

**Figure 4:**
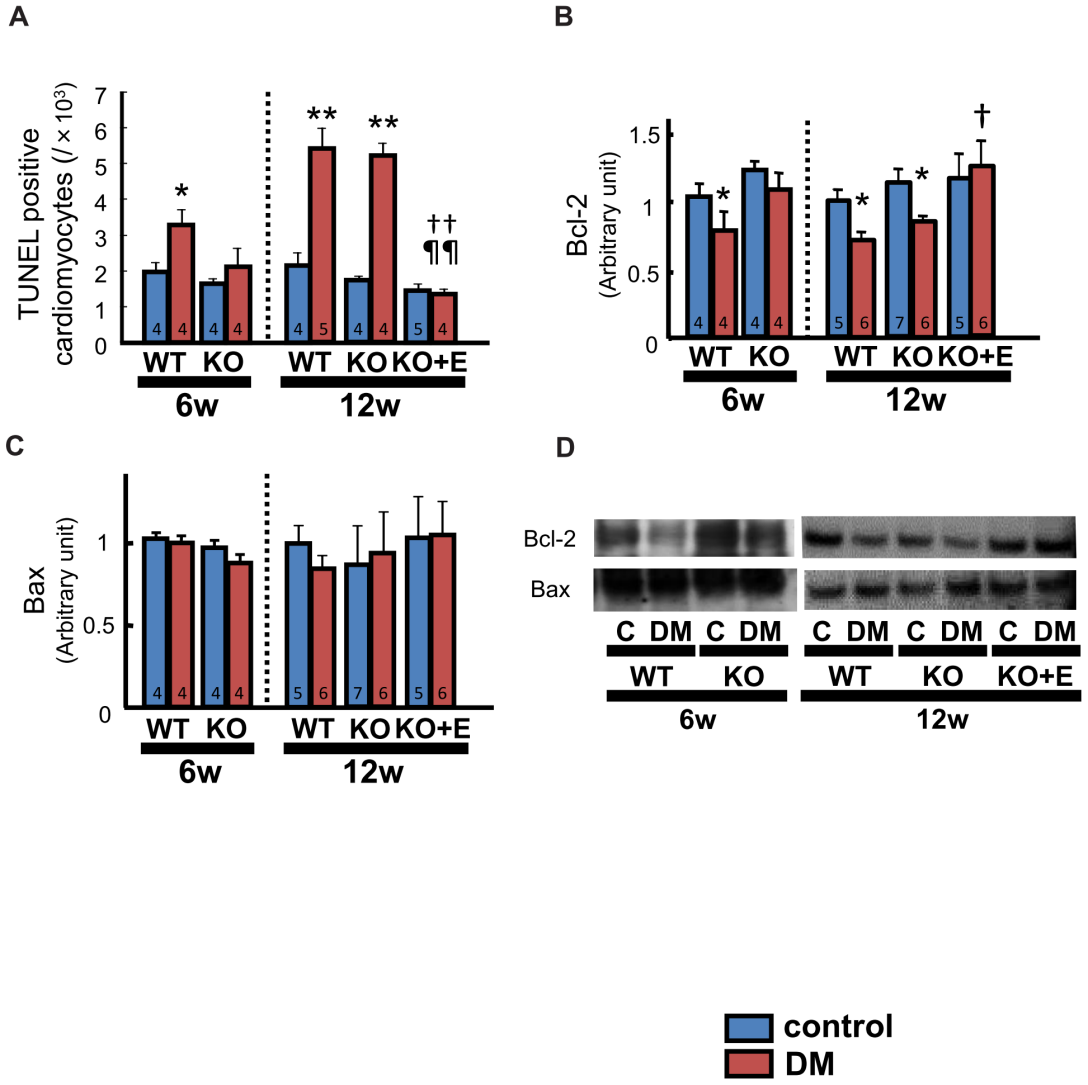
Apoptosis in the diabetic heart. The number of terminal deoxynucleotidyl transferase-mediated dUTP nick-end labeling (TUNEL)-positive cardiomyocytes in LV tissue (A), expression of Bcl-2 (B) and Bax (C) protein in LV tissue determined by Western blotting, and representative images of the bands for Bcl-2 and Bax protein (D). Number of mice per group is indicated in bar graph. Values are the mean±SEM. DM, diabetes mellitus; KO, AT_1a_ receptor knockout; WT, wild-type; C, control. *p<0.05 vs. the respective control group, **p<0.01 vs. the respective control group, †p<0.05 vs. WT-DM, ††p<0.01 vs. WT-DM, ¶¶p<0.01 vs. KO-DM.

At 6 weeks, the number of TUNEL-positive cardiomyocytes was increased in WT-DM mice compared with WT-control mice (3.37±0.40 vs. 1.97±0.27/10^3^ nuclei, p<0.05), but there was no significant difference between the KO-DM and KO-control groups (2.15±0.48 vs. 1.66±0.13/10^3^ nuclei, p = N.S.). At 12 weeks, the number of TUNEL-positive cardiomyocytes was also increased in KO-DM mice compared with KO-control mice (WT-control 2.16±0.35 vs. WT-DM 5.46±0.56/10^3^ nuclei, p<0.01; KO-control 1.77±0.08 vs. KO-DM 5.27±0.31 nuclei, p<0.01). Eplerenone treatment prevented these changes (KO-control+E 1.48±0.16 vs. KO-DM+E 1.38±0.12 nuclei, p = N.S., **Figure 4A**).

At 6 weeks, the Bcl-2 protein level was significantly lower in WT-DM mice, but it showed no significant difference between the KO groups. At 12 weeks, Bcl-2 protein was also downregulated in KO-DM mice compared with KO-control mice. Eplerenone treatment also prevented these changes (**Figure 4B**). Bax protein levels were similar among all groups at both 6 and 12 weeks (**Figure 4C**).

### Oxidative Stress

**Figure 5:**
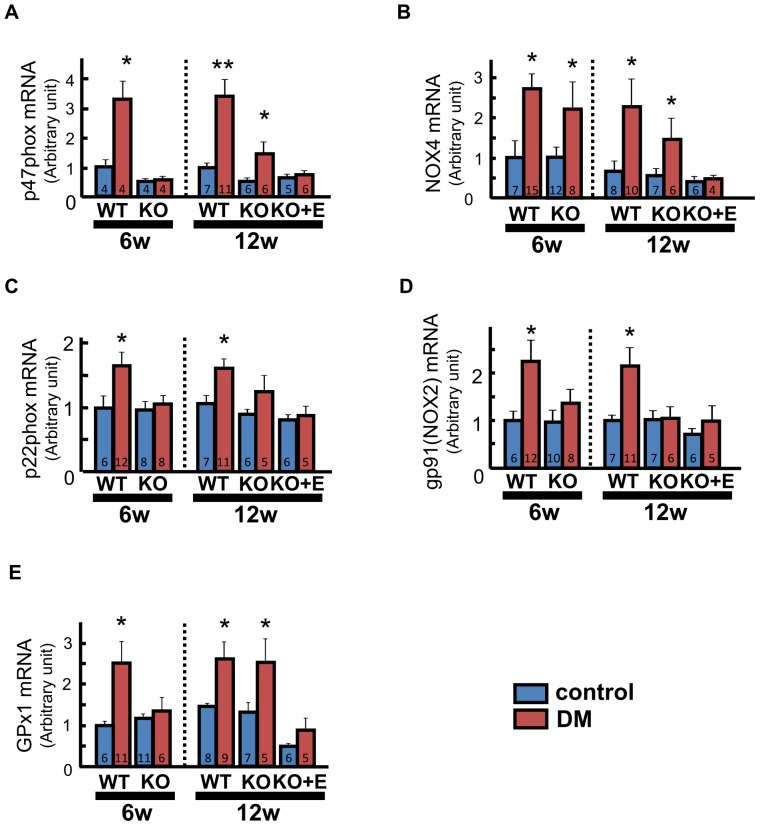
Oxidative stress in the diabetic heart. Expression of mRNAs for nicotinamide adenine dinucleotide 3-phosphate (NADPH) oxidase subunits, p47phox (A), NOX4 (B), p22phox (C), NOX2 (D), and glutathione peroxidase type 1 (GPx1) (E) in LV tissue measured by real-time PCR. Number of mice per group is indicated in bar graph. Values are the mean±SEM. DM, diabetes mellitus; KO, AT_1a_ receptor knockout; WT, wild-type. *p<0.05 vs. the respective control group.

Nicotinamide adenine dinucleotide 3-phosphate (NADPH) oxidase has an important role in the production of reactive oxygen species (ROS) [30] by catalyzing electron transfer from NADPH to molecular oxygen to form O_2_^−^. Glutathione peroxidase is an enzyme that is upregulated in response to an increase of ROS and catalyzes the reduction of hydrogen peroxide. NADPH oxidase p47phox subunit and glutathione peroxidase (GPx1) mRNA expression were significantly upregulated in WT-DM mice compared with WT-control mice at 6 weeks, whereas there was no significant difference between the KO groups. At 12 weeks, however, p47phox and GPx1 mRNA levels were also upregulated in KO-DM mice compared with KO-control mice. Eplerenone treatment prevented such changes (**Figure 5A, 5E**). NOX4 mRNA was upregulated in WT-DM and KO-DM mice compared with the respective control groups at both 6 and 12 weeks. Eplerenone prevented such changes in KO-DM mice (**Figure 5B**). Expression of p22phox and NOX2 mRNA was significantly upregulated in WT-DM mice at 6 and 12 weeks, but was similar among KO-groups at both times (**Figure 5C, 5D**).

**Figure 6:**
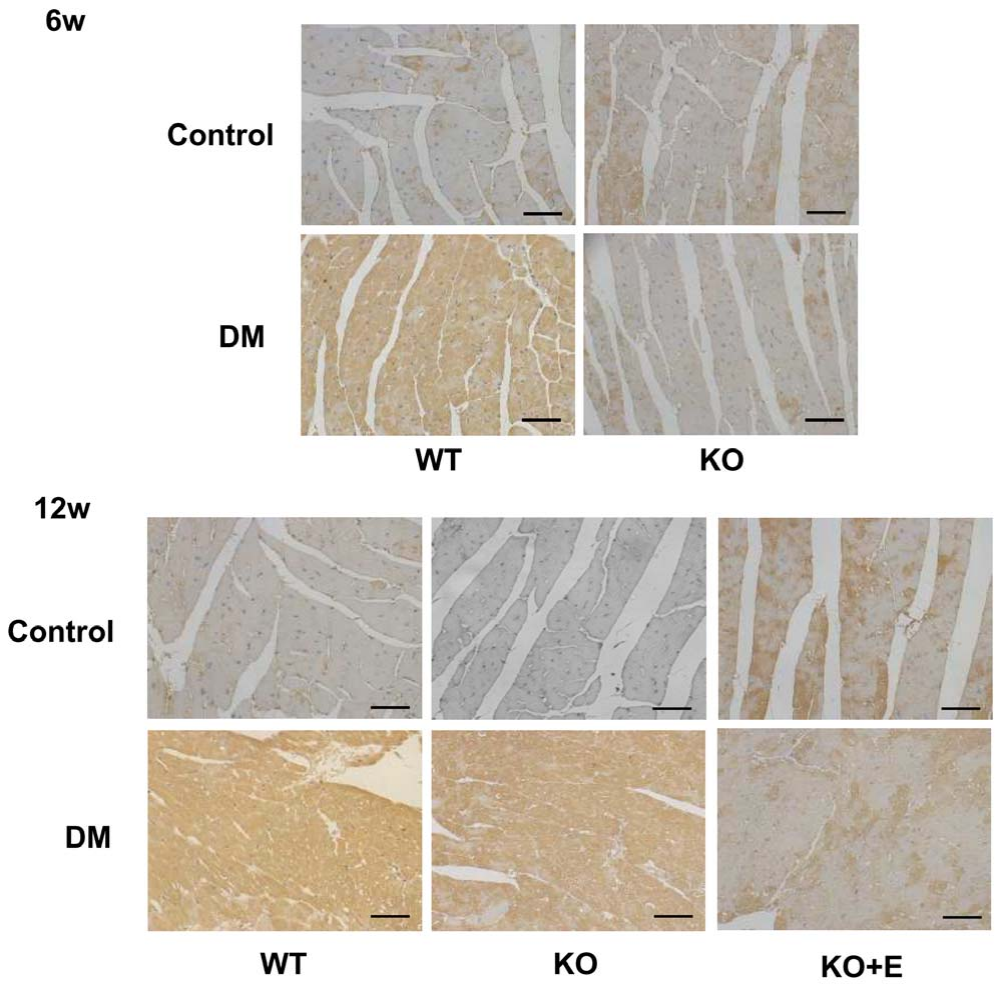
The immunohistochemistry of LV tissue for 4-hydroxy-2-nonenal (4-HNE) in the diabetic heart. Brown staining is positive for 4-HNE, while nuclei are stained blue by hematoxylin. At 6 weeks, staining for 4-HNE was stronger in WT-DM mice compared with WT-control mice, whereas there was no significant difference between KO-DM and KO-control mice. At 12 weeks, however, staining for 4-HNE was also stronger in KO-DM mice compared with KO-control mice. Eplerenone treatment prevented these changes. All pictures are at ×400 magnification. Scale bar = 50 μm.

At 6 weeks, staining for 4-HNE, a byproduct of lipid peroxidation and an index of oxidative stress [10], was stronger in LV tissue obtained from WT-DM mice compared with WT-control mice, whereas there was no significant difference between KO-DM and KO-control mice. At 12 weeks, however, staining for 4-HNE was also stronger in KO-DM mice compared with KO-control mice. Eplerenone treatment prevented these changes (**Figure 6**).

### RAAS Activation

**Figure 7:**
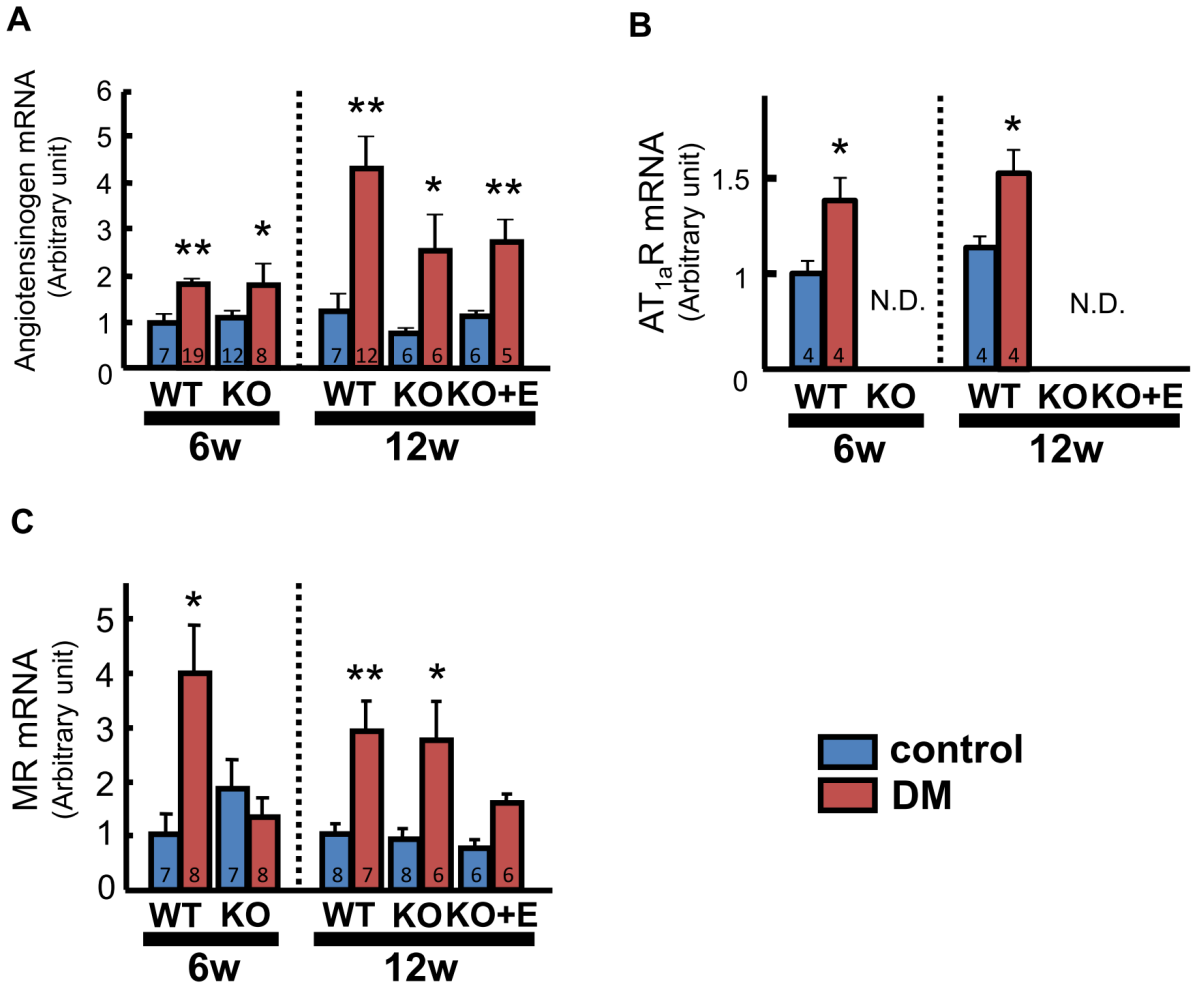
Renin-angiotensin-aldosterone system in the diabetic heart. Expression of mRNAs for angiotensinogen (A), angiotensin II type_1a_ receptor (AT_1a_R) (B), and the mineralocorticoid receptor (MR) (C) in LV tissue measured by real-time PCR. Number of mice per group is indicated in bar graph. Values are the mean±SEM. DM, diabetes mellitus; KO, AT_1a_ receptor knockout; WT, wild-type; N.D., not detected. *p<0.05 vs. the respective control group, **p<0.01 vs. the respective control group.

At both 6 and 12 weeks, angiotensinogen mRNA expression was upregulated in all of the DM groups compared with the respective control groups (**Figure 7A**). At 6 and 12 weeks, AT_1a_R expression was upregulated in WT-DM mice compared with WT-control mice (**Figure 7B**). At 6 weeks, the MR mRNA level was 3.9-fold higher in WT-DM mice compared with WT-control mice, but there was no significant difference between the KO-DM and KO-control groups. At 12 weeks, however, the level of MR mRNA was increased by 2.4-fold in WT-DM, and by 3.0-fold even in KO-DM mice. Eplerenone treatment prevented these changes (**Figure 7C**).

**Figure 8:**
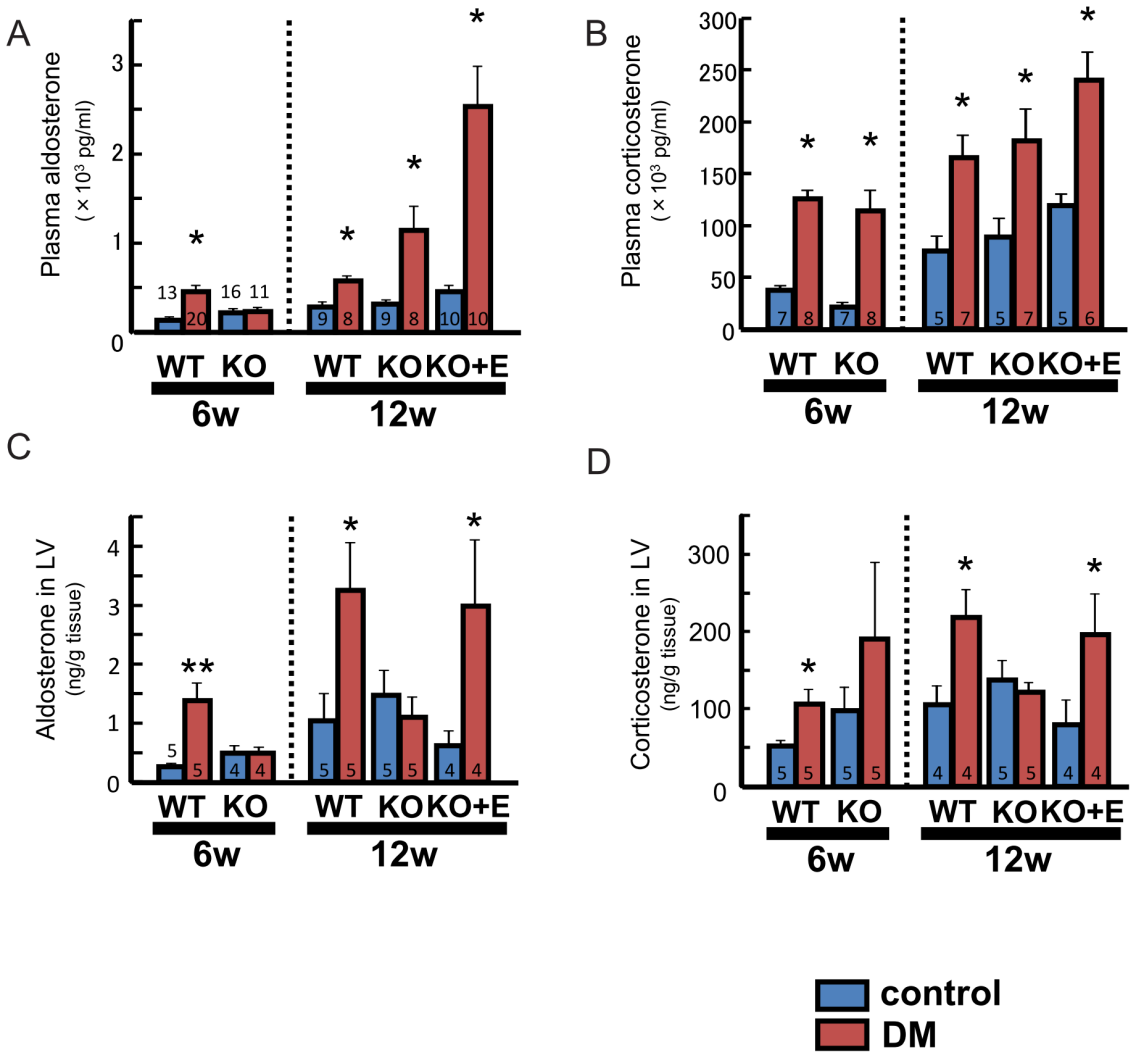
Aldosterone and corticosterone levels. Plasma aldosterone (A) and corticosterone (B) concentrations. Aldosterone content (C) and corticosterone content (D) of LV tissue. Number of mice per group is indicated in bar graph. Values are the mean±SEM. DM, diabetes mellitus; KO, AT_1a_ receptor knockout; WT, wild-type; LV, left ventricle. *p<0.05 vs. the respective control group, **p<0.01 vs. the respective control group.

PAC was elevated in WT-DM mice at 6 weeks. Although PAC was not elevated in KO-DM mice at 6 weeks, it was significantly elevated at 12 weeks, suggesting that the “aldosterone breakthrough” phenomenon had occurred (**Figure 8A**). PAC was also higher in KO-DM+E mice than KO-control+E mice. However, the aldosterone content of LV tissue was increased in WT-DM mice, but was unchanged in KO-DM mice (**Figure 8C**). That is, “aldosterone breakthrough” was not identified in the local LV tissue RAAS, unlike the systemic RAAS. The plasma corticosterone concentration was elevated in all of the DM groups (**Figure 8B**). In LV tissue from KO-DM mice, however, the corticosterone content was not significantly increased at either 6 or 12 weeks (**Figure 8D**).

## Discussion

To the best of our knowledge, this is the first report which clearly showed blockade of AT_1_ signaling could prevent, although partially, the chronic deterioration of LV function induced by DM and addition of an MR antagonist had a further cardioprotective effect.

One of the most important findings was the documentation of RAAS activation in the diabetic heart, while genetic ablation of AT_1_ signaling prevented LV diastolic dysfunction in diabetic mice at 6 weeks. By 12 weeks, however, ablation of AT_1_ signaling could not prevent DM-induced LV diastolic dysfunction. Additional interruption of MR signaling with eplerenone ameliorated these changes.

The second important finding was that aldosterone breakthrough was only demonstrated in the plasma and was not found in local LV tissue.

The third important finding was that prevention of LV dysfunction by RAAS blockade was accompanied by attenuation of potential mechanisms of LV failure such as abnormal Ca^2+^ handling, oxidative stress, and apoptosis.

### RAAS in the Diabetic Heart

Previous reports have shown local renin-angiotensin system activation in the diabetic heart [31]. Blockade of AT_1_ signaling with ACE-I or ARB therapy has been shown to reverse DM-induced cardiac dysfunction without affecting the blood glucose level [4], [32]. Although such previous studies assessed the effects of AT_1_ signaling, the role of MR signaling has not been adequately evaluated so far.

There is a growing body of evidence that the MR mediates a variety of actions in the cardiovascular system and plays an important role in the process of ventricular remodeling independent of blood volume and blood pressure [33], [34]. Transgenic mice overexpressing the human MR have a normal blood pressure, but show an increase of LV diameter, as well as systolic dysfunction [35] and a high rate of sudden death linked to severe ventricular arrhythmias, which are prevented by treatment with spironolactone [36]. MR mRNA expression was upregulated in rats with experimental hypertension [37] or MI [38], while spironolactone significantly suppressed the expression of these genes [37], [38]. Some *in vitro* studies have also shown that aldosterone significantly increases MR mRNA expression [39]. Since aldosterone production and cardiac sympathetic activity are enhanced by pathological conditions, particularly after MI, it is conceivable that these factors would increase MR expression in the myocardium, although detailed mechanism has not been clarified.

In the present study, we confirmed upregulation of the local RAAS in diabetic hearts. Such upregulation of the local RAAS in the LV of diabetic animal can induce LV dysfunction, as well as cardiomyocyte apoptosis and increased oxidative stress. Genetic RAAS blockade at the AT_1_ receptor level ameliorated these changes over the *short-term*. Interestingly, although MR mRNA expression was not upregulated in KO-DM mice at 6 weeks, it was significantly upregulated at 12 weeks (**Figure 7C**). This AT_1_-independent MR activation was associated with LV diastolic dysfunction, cardiomyocyte apoptosis, and increased oxidative stress, while the selective MR blocker eplerenone prevented these changes. Therefore, AT_1_-independent MR activation might be involved in the mechanism of the development of diabetic cardiac injury.

### “Aldosterone Breakthrough” and MR Activation in the Diabetic Heart

In 1981, Staessen *et al.* reported an initial decrease and subsequent increase of PAC in hypertensive patients treated with captopril, an ACE-I [13]. These findings were confirmed by other researchers and MR antagonists were shown to be beneficial in this setting [14], [40], [41]. The late increase of PAC is related to changes of CYP11B2 (aldosterone synthase) expression in human adrenocortical cells [12].

Several previous studies have revealed that aldosterone is produced in cardiac tissues, particularly in patients with pathological conditions such as MI [22] or HF [42], although its level is much lower than that in the blood. Previous reports have indicated that LV dysfunction occurs in experimental animals with HF in the absence of an increase of PAC, but is reversed by MR blockade [37], [43]. In those studies, little aldosterone was detected in the LV tissues. These findings provided evidence that plasma aldosterone independent MR activation in myocardium plays a role in the mechanism of LV dysfunction.

The MR has a high affinity for corticosterone and cortisol, as well as aldosterone [44]. Expression of 11β-hydroxysteroid dehydrogenase type 2 (11β-HSD2), which converts endogenous glucocorticoids to their receptor-inactive 11-keto analogues, is reported to be extremely low in the heart, indicating that cardiomyocyte MRs are primarily occupied by endogenous glucocorticoids that are present at higher levels than aldosterone [45], although the 11β-HSD2 mRNA expression was found to be upregulated in rats with MI [38]. Although glucocorticoids generally antagonize the MR and act as cardioprotective hormones in nonepithelial cells such as myocytes [46], it has been shown that glucocorticoid effects are modulated by the redox state of cells and that glucocorticoids activate MR signaling in an oxidized state [44].

Although plasma aldosterone independent MR activation in myocardium play a part in the development of MI remodeling and HF, it is not clear whether the same mechanism is involved in the development of diabetic heart injury. Therefore we focused on *plasma* and *local* contents of aldosterone and corticosterone, and MR activation in myocardium with diabetic animals.

The present study demonstrated that expression of MR mRNA in the LV was upregulated in WT-DM mice at 6 weeks and even in KO-DM mice at 12 weeks (**Figure 7C**). In addition, PAC was increased in WT-DM mice at 6 week and also in KO-DM mice at 12 weeks (aldosterone breakthrough, **Figure 8A**). It seems the change of MR mRNA expression was parallel to those of PAC. However, the aldosterone or corticosterone content of the LV was not increased in KO-DM mice (**Figure 8C, 8D**). Collectively, these findings suggest that, in this setting, MR signaling is regulated separately from aldosterone or corticosterone in the myocardium, although *plasma* aldosterone may show a “breakthrough”. We could not find any intergroup differences of 11β-HSD2 and CYP11B2 mRNA expression in the LV (data not shown). These findings suggested that the mechanism of MR activation in diabetic hearts did not contain local aldosterone production in the myocardium. The present *in vivo* experiments did not allow us to determine the precise molecular mechanisms by which MR signaling was activated, so further studies are needed to address these issues.

### Ca^2+^ Handling Abnormality in the Diabetic Heart as a Potential Mechanism of LV Relaxation Failure

We found that LV relaxation was impaired in diabetic hearts. And the data regarding the components involved in Ca^2+^ handling changed in parallel with the markers of LV relaxation and MR activation. From the present findings, RAAS activation contributed to LV diastolic dysfunction and abnormal Ca^2+^ handling. Previous reports have indicated that the mechanism of impaired LV relaxation involves disturbance of Ca^2+^ handling [7]. In fact, adding SERCA2a to mice with STZ-induced diabetes normalizes both systolic and diastolic function [47]. Also, the Ca^2+^ transient had a slower time to peak and slower decay in LV trabeculae from diabetic rats, along with a slower time course of contraction [48]. Changes of SERCA2a expression and phosphorylation of phospholamban might be responsible for these alterations of Ca^2+^ transients and could play an important role in the occurrence of LV diastolic dysfunction in diabetic hearts, although there are many known factors that influence diastolic function. Collectively, although these findings including previous publications support our suggestion as to potential mechanism of diabetic cardiomyopathy regulated by RAAS, further evaluations are necessary to confirm the detailed mechanism.

### Oxidative Stress and Apoptosis in the Diabetic Heart as a Potential Mechanism of LV Failure

In our data, the markers of oxidative stress and apoptosis changed in parallel with the data of LV function and MR activation. The findings of the present study (**Figure 4** **–** **6**) suggest that ROS production and cardiomyocyte apoptosis are enhanced in the diabetic heart, at least partly due to activation of the local RAAS, and contribute to the pathogenesis of diabetic cardiomyopathy. Previous studies have shown that apoptosis is accelerated in various organs of DM patients including the heart, which is associated with increased oxidative stress [9], [10]. Both expression of some RAAS components and apoptosis were enhanced in cardiomyocytes from diabetic mice, while treatment with an ARB inhibited such changes and prevented cardiomyocyte apoptosis [9].

The findings in the present study are consistent with these reports and suggest the possibility that not only AT_1_ but MR activation is also involved in these mechanisms, as shown in experimental MI model [15], [43].

### Clinical Implications

This study showed that AT_1_ blockade alone protects the heart from damage caused by DM over the *short-term*, but it is necessary to block both AT_1_ and MR signaling over the *long-term*. These findings are consistent with the results of large-scale clinical trials, RALES [16], EPHESUS [17], EMPHASIS-HF [18] and Aldo-DHF study [19], even though the subjects of those studies were patients with HF or MI. In a subanalysis in EPHESUS, the prognosis of diabetic patients with HF following MI was improved by RAAS inhibition through combined therapy with an ACE-I/ARB and MR antagonist [49]. This beneficial effect of RAAS inhibition can be at least partly explained by its protective effect on the heart, and may suggest a strategy for prevention of cardiac complications in diabetic patients.

### Limitations

In the present study LV systolic pressure was significantly lower in the WT-DM and KO-DM groups compared with the respective control groups at 12 weeks (**Figure 2C**). One can say the data which represent LV systolic and diastolic function could be influenced by lower blood pressure in DM mice compared with control at 12w. Actually LV systolic pressure was reduced in DM mice at 12 week and it is possible that the deterioration of LV functional indices might reflect it. However, at 6 week Vcfc was already decreased in WT-DM irrespective of no significant difference in blood pressure (**Figure 2B, 2C**). The changes in these indices corresponded to the changes in molecular analysis such as Ca^2+^ handling (SERCA2a expression, phosphorylated phospholamban/total phospholamban ratio) or apoptosis (TUNEL staining). Previous studies showed the disturbance of systolic function in diabetic heart in rodents [5]. Taken these findings together we consider that the decrease of systolic blood pressure might be caused by deterioration of LV contractility. However, it is the limitation that we could not draw the logical conclusion on this issue from the data obtained in the present study since we did not measure the indices that represent LV contractility independent of blood pressure such as end-systolic elastance (Ees) [50].

We used mice with STZ-induced type 1 DM in the present study. However, the prevalence of type 2 DM is much higher than that of type 1 DM, and the present study did not allow us to assess the role of insulin resistance. These are considered to be limitations of the present study.

In conclusion, pathological activation of the RAAS, especially AT_1_ independent MR activation, plays a crucial role in the development of DM-induced LV dysfunction. AT_1_ blockade alone might be insufficient and additional MR blockade could be important for preventing the onset and development of diabetic cardiomyopathy.
